# Noninvasive Calculation of Cerebrospinal Fluid Production Using Phase-Contrast Magnetic Resonance Imaging: First Implementation in Augusta, Georgia

**DOI:** 10.7759/cureus.39686

**Published:** 2023-05-30

**Authors:** Tucker Oliver, Samuel Macomson, Bruce Gilbert, Scott Forseen

**Affiliations:** 1 Neurological Surgery, Augusta University Medical College of Georgia, Augusta, USA; 2 Radiology, Augusta University Medical College of Georgia, Augusta, USA

**Keywords:** csf, noninvasive, production, aqueduct, phase-contrast mri, cerebrospinal fluid

## Abstract

This retrospective review examines the utility of phase-contrast magnetic resonance imaging (PC-MRI) to calculate flow through the aqueduct between the third and fourth ventricles to estimate cerebrospinal fluid (CSF) production. Imaging software quantified the CSF flow rate across the aqueduct of four females and two males at a single center, and the mean of these results was compared to the established mean CSF production calculated by invasive techniques. There was no significant difference between the means, contributing to the body of literature suggesting the utility of PC-MRI in estimating CSF production rates.

## Introduction

Cerebrospinal fluid (CSF) is critical to maintaining the central nervous system (CNS). According to Cushing’s "third circulation" description, CSF is produced mainly by the choroid plexus in the ventricles and exits by the foramen of Luschka and Magendie to fill the subarachnoid space and disperse to the rest of the CNS [1]. In addition to providing buoyancy and protection for the brain, it also delivers metabolites and clears waste. Recent studies of CSF continue to reveal new avenues of functionality and importance [2-4]. Invasive methods have established an accepted average production rate of 0.3-0.4 mL/min, with a total CSF volume of 90-150 mL in adult humans [5]. However, varying results from a variety of methods of measurement in distinct disease states continue to generate debate and diminish the validity of applying the average adult CSF production rate to the individual patient [6-8]. These findings establish the need to accurately quantify CSF production in the indicated patients while minimizing invasiveness. Phase-contrast magnetic resonance imaging (PC-MRI), which calculates CSF flow across the cerebral aqueduct, is a noninvasive estimate of overall CSF production [9]. In this prospective observational study, we review calculations of the CSF flow across the aqueduct in several patients using PC-MRI at a single institution and compare these to the established average production rate.

## Materials and methods

Population and study design

Patients were selected for the study from the outpatient elective MRI schedule. The selected patients presented with indications for elective routine brain MRI scans. The patients were notified by phone prior to the day of their scheduled MRI and asked if they would be willing to participate in the study. Upon arrival for the MRI scan, the patients consented to an additional phase-contrast series and thin-cut high-resolution axial sections along the length of the cerebral aqueduct. The study was approved by the Augusta Institutional Review Board (IRB), Augusta, Georgia. Enrollment took place between November 2019 and January 2020. Inclusion criteria were patients scheduled for elective MRI scans of the brain between November 2019 and January 2020. Exclusion criteria included a history of hydrocephalus or pathology requiring shunt placement and contraindications to MRI scanning, including claustrophobia, anxiety, or the presence of metal implants or devices in the body. Eight patients consented and were scanned, but two patients were excluded from the data due to corrupted images. The scans of four females and two males were evaluated, and the average age was 25.7 years. Two patients received scans to trend lesional pathology; three patients were evaluated for seizures; and one patient presented with nocturnal vomiting. Patients did not undergo invasive testing.

Imaging technique and data calculation

For each patient, the phase-contrast time sequence was recorded across a single axial MRI slice. Imaging software Phillips IntelliSpace portal Q-flow analysis quantifies the flow rate across the cross-section of the cerebral aqueduct, identified by a user-defined polygonal outline. The area of interest along the aqueduct was chosen by a board-certified neuroradiologist with expertise in phase-contrast MRI interpretation. The peak velocity was extrapolated from the sequence and used as the velocity factor in the flow rate equation. The radius of the aqueduct was measured from the separate, high-resolution axial MRI cut at the same level to find the area of the circle formed by the two-dimensional cut. The flow rate of CSF (cm/sec) was multiplied by the area of the circle (cm2) to calculate volumetric CSF flow (cm3/min) and estimate production.

Statistical analysis

A two-tailed, one-sample t-test was used to compare the mean calculated CSF production rate of these data (n=6) with the mean CSF production rate calculated via invasive methods by Rubin et al. [5]. The level of significance was set at p=0.05.

## Results

Eight patients were enrolled in the study, but two were excluded from the data collection due to corrupted data obtained in the imaging process. Each study patient was scanned on the same Philips 3 Tesla (3T) magnetic resonance imaging scanner. The brain MRI scans of the four female and two male study participants were individually evaluated by a board-certified neuroradiologist with expertise in phase-contrast MRI. The average age of the participants enrolled in the study was 25.7 years. The ages ranged from six months old to 76 years old. One study participant had a known meningioma along the convexity and did not have a connection to the ventricular system. Another participant had been diagnosed with an incidental arachnoid cyst that was being followed for interval changes. The arachnoid cyst was located in the middle cranial fossa and had no communication with the ventricular system. A third study participant was evaluated with an MRI for nocturnal vomiting, and the MRI did not reveal any abnormal findings. Three study participants were evaluated with an MRI for new-onset seizure activity. No abnormal intracranial pathology was identified in any of these three patients. The mean radius, in centimeters, of the cerebral aqueduct was 0.097, ranging from 0.075 cm to 0.120 cm. The mean area of the aqueduct, calculated by using the equation for the area of a circle, A = pi r2, was 0.030 cm2. The mean area of the aqueduct ranged from 0.018 cm2 to 0.045 cm2. The mean calculated peak velocity was 10.0662 cm/s. The calculated velocities ranged from 8.220 cm/s to 16.540 cm/s (Table 1).

**Table 1:** Patient demographics and calculated flow rates from PC-MRI

Patient ID#	Age (years)	Sex	Indication for MRI	Radius (cm)	Area (cm^2)	Peak velocity (cm/s)	Flow (cm^3^/s)
1	76	Female	Meningioma	0.115	0.042	12.180	0.506
2	9	Male	Nocturnal vomiting	0.095	0.028	16.540	0.469
3	51	Female	Arachnoid cyst	0.075	0.018	8.520	0.151
4	8	Male	Seizures	0.075	0.018	8.220	0.145
5	6 months	Female	Seizures	0.100	0.031	9.290	0.292
6	8	Female	Seizures	0.120	0.045	9.220	0.417
Mean	25.7			0.097	0.030	10.662	0.330

The CSF flow rate across the cerebral aqueduct for these six patients was calculated using the fluid dynamics flow rate equation Q=vA. We hypothesized that if the CSF flow rate in these patients was calculated by PC-MRI, there would be no significant difference between the mean flow rate of these patients and the mean flow rate of the lumbar-subarachnoid perfusion study, cited as the most accurate calculation of invasive CSF production by Rubin et al. The PC-MRI data were also compared for completeness to the interventricular perfusion study, which has been noted as inaccurate for not including extra-ventricular production by Rubin et al. [5]. The mean calculated CSF flow rate was 0.330 cm3/sec by PC-MRI. The calculated flow rate ranged from a low of 0.145 cm3/s to 0.506 cm3/s. Compared to the lumbar-subarachnoid perfusion study, the result of the two-tailed, one-sample t-test failed to reject the null hypothesis with a p-value of 0.55, suggesting that there is not a statistically significant difference. The null hypothesis was rejected for the ventricular perfusion study, with a p-value of 0.02, suggesting a statistically significant difference (Table 2).

**Table 2:** Flow rate comparison *Two-tailed, one-sample t-test †From Rubin et al. [5]

Zero-outflow pressure tests†	Number of patients	Age (years)	Average age (years)	Number of data points	Mean flow (cm^3^/s)	p-value
Lumbar-subarachnoid perfusion	3	12, 14, 50	25.3	8	0.37	0.55*
Interventricular perfusion	5	43, 43, 51, 51, 53	48.2	20	0.40	0.02*

## Discussion

Initial modeling of cerebrospinal fluid (CSF) production in rabbits in 1963 compared relative cell volumes between the choroid plexus venous blood and aortic arterial blood [10]. The method required invasive cortical resection and underestimated CSF production rates due to decreased intracranial pressure [9,11]. In 1966, Rubin et al. performed the first study of CSF production in humans by perfusing a ventricle with inulin and monitoring clearance from either another ventricular site or the lumbar spine. The average CSF production rate from the lumbar perfusion series was 0.37 mL/min, and the average rate from the isolated ventricular system by varying avenues was 0.40 mL/min. The lumbar perfusion series is noted to likely account for CSF drained from the total ventricular and subarachnoid spaces and is a more accurate measurement of total CSF production. Due to their invasiveness, the methods of examining perfusion via lumbar puncture and ventriculostomy described by Rubin et al. were limited in humans to intraoperative study in cases of neoplasms [5].

PC-MRI sequences, first described in 1986, use a combination of phase contrast injection and MRI to generate a distinction between stationary and mobile hydrogen nuclei and quantify the direction and velocity of blood flow [12]. It was soon modified for CSF circulation in awake humans by targeting flow across the aqueduct connecting the third and fourth ventricles, and it is used in the workup of hydrocephalus [13]. Although the dural venous plexus likely plays a role in CSF absorption, especially in infants [14], the majority of the total CSF produced flows through the cylindrical aqueduct [1]. Therefore, the flow rate in these models is roughly equilibrated with total CSF production.

The average CSF flow rate across the aqueduct in this single-center study was not significantly different from the average flow rate from the lumbar perfusion series, contributing to the growing body of literature suggesting that PC-MRI could serve as an accurate noninvasive method to measure CSF production for these patients.

There are disadvantages inherent in using still images to measure a dynamic system. PC-MRI does not account for CSF produced in the fourth ventricle or from extra-choroidal sources. Given its brief window of capture, PC-MRI is also limited in establishing an average flow rate over an extended period of time, which can reduce its accuracy in cases of dynamic flow and production rate changes due to physiologic processes or anatomical features [9]. Net CSF flow increases during expiration and reverses with inspiration [15,16]. Endoscopic inspection of the aqueduct revealed the potential for variation in the accepted cylindrical shape [16]. New studies have also challenged the traditional understanding of flow dynamics. Lindstrom et al. suggested that CSF exhibits retrograde flow in cases of idiopathic normal-pressure hydrocephalus [17].

Limitations

This study is limited by its sample size of six patients. The flow rate obtained from the aqueduct is also the only data point obtained for each patient in a single sitting. Therefore, it is not known if these estimates would be consistent if compared to additional scans or if the production rates were calculated using other methods.

## Conclusions

A reliable, non-invasive measurement of CSF production could be a valuable tool in determining the underlying causes of many pathological conditions within the central nervous system. Invasive methods performed over fifty years ago quantified an average CSF production rate in humans that is generally extrapolated to patients, despite being measured in patients with mass lesions. Recent studies of CSF composition and flow dynamics, combined with advances in imaging, have cultivated an interest in quantifying CSF dynamics in the individual patient. This single-center study suggests that phase-contrast magnetic resonance imaging, although somewhat limited by temporal, spatial, and physiologic factors, shows promise as a safe, non-invasive tool to measure CSF flow through the aqueduct and approximate CSF production.
